# Optimization and Quality Evaluation of the Interlayer Bonding Performance of Additively Manufactured Polymer Structures

**DOI:** 10.3390/polym12051166

**Published:** 2020-05-19

**Authors:** Patrick Striemann, Daniel Hülsbusch, Michael Niedermeier, Frank Walther

**Affiliations:** 1 Laboratory of Material Testing, University of Applied Sciences Ravensburg-Weingarten, Doggenriedstraße 42, D-88250 Weingarten, Germany; niedermeier@rwu.de; 2 Department of Materials Test Engineering (WPT), TU Dortmund University, Baroper Str. 303, D-44227 Dortmund, Germany; daniel.huelsbusch@tu-dortmund.de (D.H.); frank.walther@tu-dortmund.de (F.W.)

**Keywords:** additively manufactured polymer structures, material extrusion, computed tomography, void distribution, infrared preheating, interlayer tensile strength

## Abstract

The application of additive manufacturing changes from prototypes to series production. In order to fulfill all requirements of series production, the process and the material characteristics must be known. The machine operator of additive manufacturing systems is both a component and a material producer. Nevertheless, there is no standardized procedure for the manufacturing or testing of such materials. This includes the high degree of anisotropy of additively manufactured polymers via material extrusion. The interlayer bonding performance between two layers in the manufacturing direction z is the obvious weakness that needs to be improved. By optimizing this interlayer contact zone, the overall performance of the additively manufactured polymer is increased. This was achieved by process modification with an infrared preheating system (IPS) to keep the temperature of the interlayer contact zone above the glass transition temperature during the manufacturing process. Combining destructive and non-destructive testing methods, the process modification IPS was determined and evaluated by a systematic approach for characterizing the interlayer bonding performance. Thereby, tensile tests under quasi-static and cyclic loading were carried out on short carbon fiber-reinforced polyamide (SCFRP). In addition, micro-computed tomography and microscopic investigations were used to determine the process quality. The IPS increases the ultimate interlayer tensile strength by approx. 15% and shows a tendency to significantly improved the fatigue properties. Simultaneously, the analysis of the micro-computed tomography data shows a homogenization of the void distribution by using the IPS.

## 1. Introduction

Manufacturing processes such as Fused Deposition Modeling (FDM™) or Fused Filament Fabrication (FFF) are based on material extrusion and generate the components layer by layer. By exploiting process-specific benefits like function integration or the production of bionically optimized structures, a holistic improvement of components is possible. Nowadays, these additive manufactured components are used as prototypes and final products. The mechanical properties represent a major challenge for the use of additively manufactured (AM) serial components. The current literature shows an anisotropic material behavior of polymers fabricated via material extrusion as well as reduced mechanical properties compared to competing production methods like injection molding [1,2,3]. The obvious weakness of AM polymer was clearly identified in the interlayer contact zone in the manufacturing direction z [3]. Thus, the overall performance of constructions depends essentially on the interlayer tensile strength. The optimization of this interlayer contact zone regarding stiffness and strength leads to an increase in the overall performance of AM polymer via material extrusion [4]. Furthermore, this is an opportunity for reducing the anisotropic material behavior [5]. Sun et al. [6] investigated different mechanisms of bond formation for material extrusion-based polymers and highlighted a thermal dependency of the interlayer bonding performance. Kousiatza and Karalekas [7] integrated a FaserBragg-grating in an FFF component. The in situ detection of temperature distribution ensures a detailed description of temperature variation within the component during the manufacturing process. Thus, the influence of temperature on the interlayer bonding and material performance was visible. Costa et al. [8] developed a temperature model for material extrusion-based manufacturing processes and highlighted a correlation between the thermal prehistory and the interlayer bonding performance. Putting this literature into context, the higher surrounding temperature results in an increased material performance, in particular for the interlayer tensile strength.

**Figure 1:**
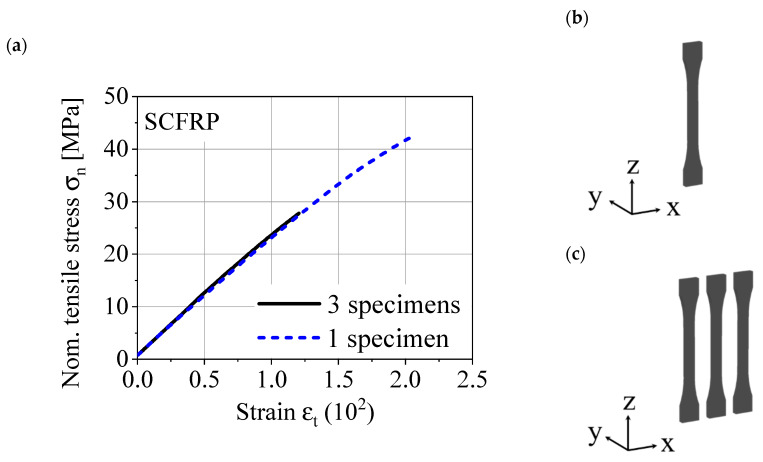
(**a**) Stress–strain diagram for short carbon fiber-reinforced polyamide (SCFRP) specimens, (**b**) 1 ZXY specimen in printing chamber, and (**c**) 3 ZXY specimen in printing chamber.

In Figure 1, a stress–strain diagram out of quasi-static tensile tests is shown. In each case, a specimen in the ZXY orientation was tested, one specimen was manufactured individually in the printing chamber and the other was manufactured with identical process parameters with two further specimens in the printing chamber. The experimental setup enforces different surface temperatures of the polymer, especially in the interlayer contact zone. The results show a significantly reduced interlayer tensile strength and highlight the challenges for a reproducible manufacturing process. Thus, the material performance of the AM component is increased by a successful diffusion process in the interlayer contact zone. This is ensured by keeping the temperature of the interlayer contact zone above the glass transition temperature of the polymer [9].

A reproducible manufacturing process requires a defined window of viscosity and shear rate for processability. This window can be adjusted by adding additives [10] or by increasing the temperature. The thermal energy for annealing the AM polymer interlayer contact zone can be applied in a post process [11] or by increasing the surrounding temperature. Both improve the mechanical properties and the reproducibility of the manufacturing process. The higher thermal energy in the interlayer contact zone increases the melt diffusion und optimizes the mechanical properties. Furthermore, the misalignment of the carbon fibers along the moving direction of printing head reinforces the interlayer contact zone [12]. There are several approaches for implementing a higher surrounding temperature. One of the most common approaches is the use of a heated printing chamber. Kishore et al. [13] achieved a significant increase of the interlayer bonding performance by preheating the surface temperature with infrared heat. Thereby, the interlayer bonding performance was determined by a double cantilever bending test in fracture mode I. Du et al. [14] introduced a numerical model for the analysis of the thermal distribution of FDM using a laser-based preheating system. Using the laser-based preheating system, the experimental results show an improvement of the interlayer bonding performance, which is characterized by the interlayer tensile strength. As for the higher surrounding temperature, there are many alternatives to characterize the interlayer bonding performance. Kishore et al. [13] and Spoerk et al. [15] used a double cantilever test in fracture mode I to determine the interlayer strength. Davis et al. [16] investigated the fracture toughness of individual welds in fracture mode III. Both experimental setups are difficult to use under cyclical loads. Sun et al. [6] characterized the interlayer bonding performance by 3-point bending tests, and Dutra et al. [17] exploited the short beam bending test according to the interlaminar shear strength for composite materials. The experimental setups with a flexural load combine the tensile and compressive stresses in the material. Du et al. [14] carried out the interlayer bonding performance as many others have with the interlayer tensile strength [18,19]. Watschke et al. [20] adapted several approaches for characterizing a multi-material interface. The load types are separated for tensile, shear and compressive loads to enable a detailed characterization of the interface.

In this study, the interlayer tensile strength is used to characterize the interlayer bonding performance. An innovative approach for quality evaluation shows the void distribution depending on the manufacturing direction z. As an example, this test methodology is demonstrated on an optimized process by an in situ infrared preheating system (IPS). Due to the large number of testing methods, the aim of this study is to establish a systematic approach for determining the interlayer bonding performance and evaluating the quality of the interlayer contact zone. The objective question that needs to be answered is whether the methodology can detect the process-related errors.

## 2. Material and Manufacturing

A short carbon fiber-reinforced polyamide (SCFRP) (CarbonX™ Nylon Gen. 2, 3DXTECH, Grand Rapids, MI, USA) was used for the investigations. The filament has a fiber weight content of approx. 12.5 wt.%, the fiber diameter is 7 μm and the fiber length distribution is 150 to 400 μm after extrusion to filament. The composite material is supplied in vacuum-packed spools with a diameter of 1.75 ± 0.05 mm. Before processing, the material was dried for 4 h at 50 °C in an oven (FP 115, Binder). Table 1 gives the different material properties of the SCRFP from previous studies. As shown in Figure 1, the results show a high degree of variation due to different parameter settings and experimental setups. In addition, the anisotropic material behavior is highlighted by significantly reduced mechanical properties by changing the built orientation in the printing chamber (XYZ, YXZ and ZXY).

**Figure 2:**
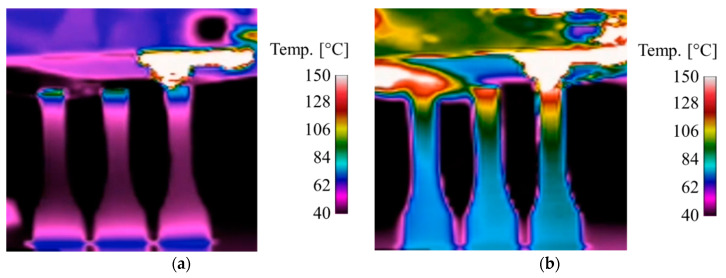
Thermographic distribution during the manufacturing process: (**a**) FFF and (**b**) FFF IPS.

The specimens were generated layer-by-layer with the additive manufacturing process FFF. In this study, the specimens were manufactured in the ZYX orientation according to ASTM F2921-11. Each print job contained three specimens in the printing chamber in order to compensate for thermal gradients during the manufacturing process, with only the same specimen per print job used for mechanical testing. Selected manufacturing parameters are displayed in Table 2. In order to evaluate the influence of IPS, standardized FFF specimens were used as a reference. The IPS consists of two ceramic radiators (QFE, Ceramix Ltd., Ballydehob, Ireland) with 150 W each and emitted long-wave infrared rays with wavelengths of 2 to 10 µm. For the selective temperature control of the current layer, the radiations are focused by additional reflectors. Figure 2 shows the standard process FFF and the modified process FFF IPS during the manufacturing process by a thermographic image. The images show, in the upper area, the hot-end for applying the molten thermoplastic material and the three upright specimens on the printing bed. The FFF IPS shows a significantly increased temperature level of the entire specimens, especially at the deposition point near the hot-end.

## 3. Experimental Setup

The quasi-static tensile tests were executed on a universal testing system (Zwick 1464, F_max_ = 50 kN) according to DIN 527. Strain measurement was done by a tactile extensometer (MultiXtens, ZwickRoell, Ulm, Germany). After preloading with +10 N, the displacement velocity was set to 1 mm min^−1^ to determine Young’s modulus in the range between 0.05% to 0.25% of strain. After that, the displacement velocity was increased to 50 mm min^−1^ to identify the tensile strength. The stop criterion was defined with a 50% drop in force.

**Figure 3:**
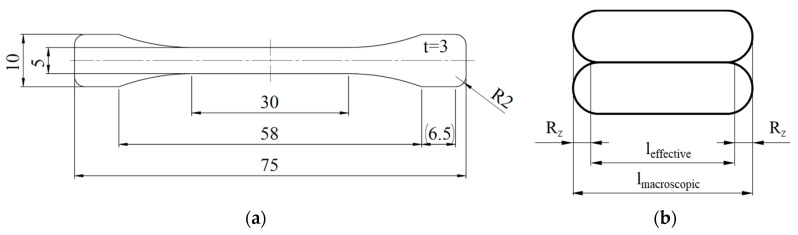
(**a**) Specimen geometry with dimensions in mm according to DIN 527-2 type 1BA, (**b**) Sketch of the cross-section correction by surface roughness R_z_ not to scale.

**Figure 4:**
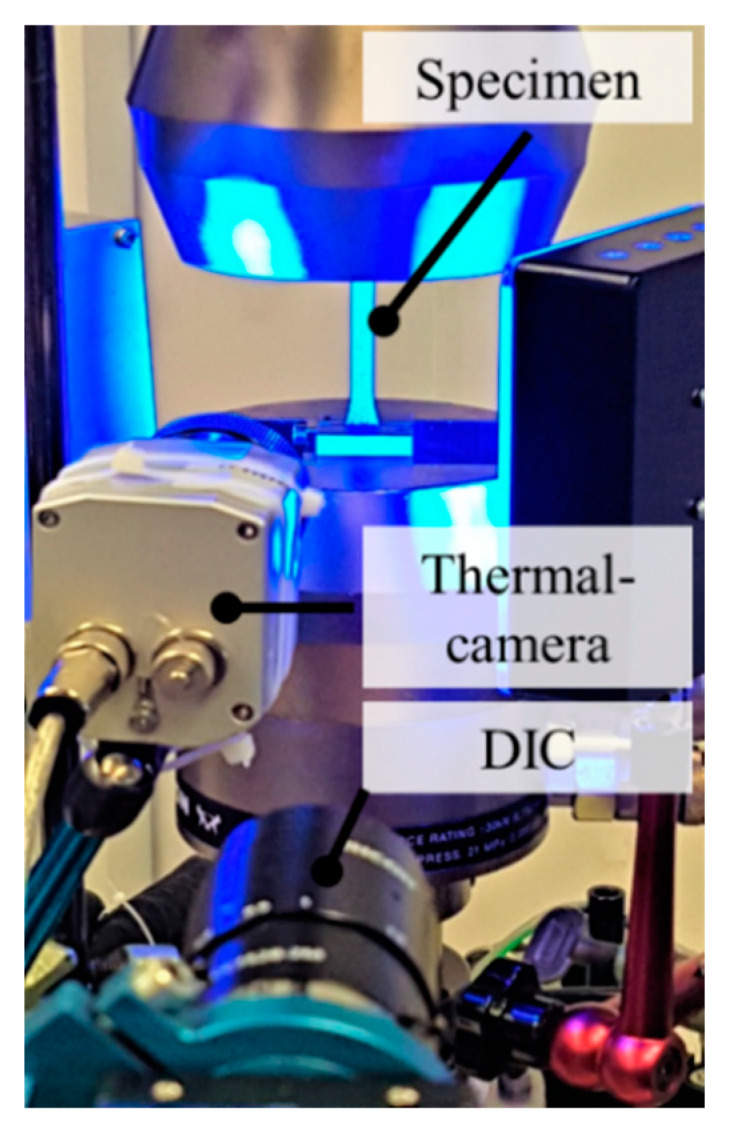
Experimental setup for destructive testing under cyclic loading.

The cyclic investigations under tensile loading were done with a servo-hydraulic testing system (Instron, 8872, F_max_ = ±10 kN) under sinusoidal tension loading with a stress-ratio R = 0.1 and a testing frequency f = 5 Hz. The deformation behavior was observed with digital image correlation (DIC) (Q-400, Limess), and the change in temperature was monitored via thermal camera (TIM 450, Micro-Epsilon, Ortenburg, Germany). For all destructive testing methods, the specimen geometry in Figure 3a was used in accordance with DIN 527. The experimental setup for the cyclic investigations is shown in Figure 4.

The microscopic investigations were performed with a 3D laser scanning confocal microscope (VK-X100, Keyence, Osaka, Japan). The optical measurement of the surface roughness was performed with a 10× lens on the untreated surfaces. The indicated surface roughness values represent the mean value of 100 profile measurements. The void analysis was carried out on the basis of micro-computed tomography (CT) scans taken by a universal micro-CT inspection system (XT H 160, Nikon, Tokyo, Japan).

## 4. Results and Discussion

In general, the results of the destructive testing methods are stress-based. The proper determination of the specimen cross-section is sophisticated due to a high macroscopic waviness in the untreated AM surface. Therefore, the specimen cross-section on which the specified stress is based was determined as follows. The macroscopic dimensions of the cross-sectional area were determined with a tactile micrometer. These macroscopic results for the length *l* and width *w* were corrected by the microscopically measured surface roughness R_z_. The sketch in Figure 3b serves as an example for the cross-section correctness of the length *l* with the surface roughness.

A_effective_ = (*w* − (2 × R_z_)) × (*l* − (2 × R_z_)), (1)

**Figure 5:**
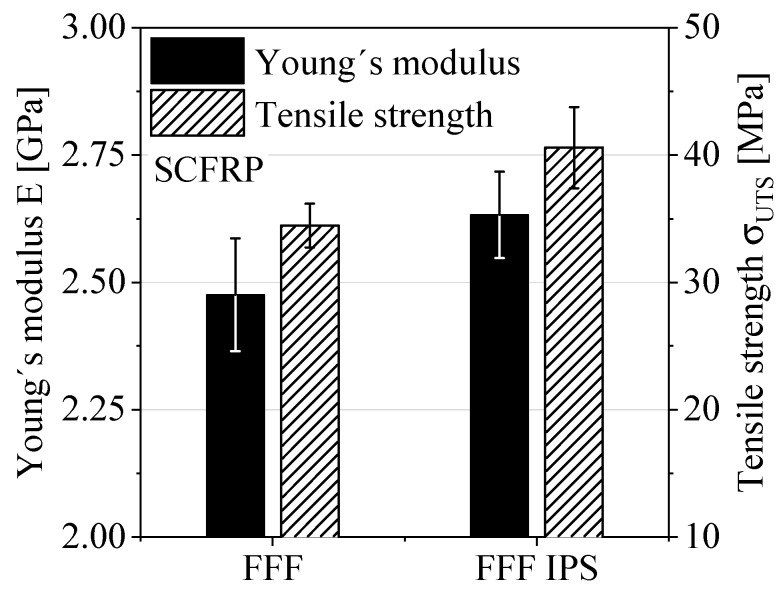
Results of the quasi-static tensile tests in terms of Young’s modulus and the tensile strength.

Because no significant differences in the surface roughness profiles were detected, an average value for R_z_ = 125 µm was used to correct all specimens. Figure 5 shows the results and corresponding standard deviations of the quasi-static tensile tests in terms of Young’s modulus and the tensile strength. The number of specimens was at least n = 5. The bar chart highlights the change in material performance caused by using the IPS. The change in Young’s modulus of about 6% in combination with the corresponding standard deviations can be considered as a tendency. The tensile strength increases by about 17%, which indicates an improvement in material performance due to the usage of IPS.

**Figure 6:**
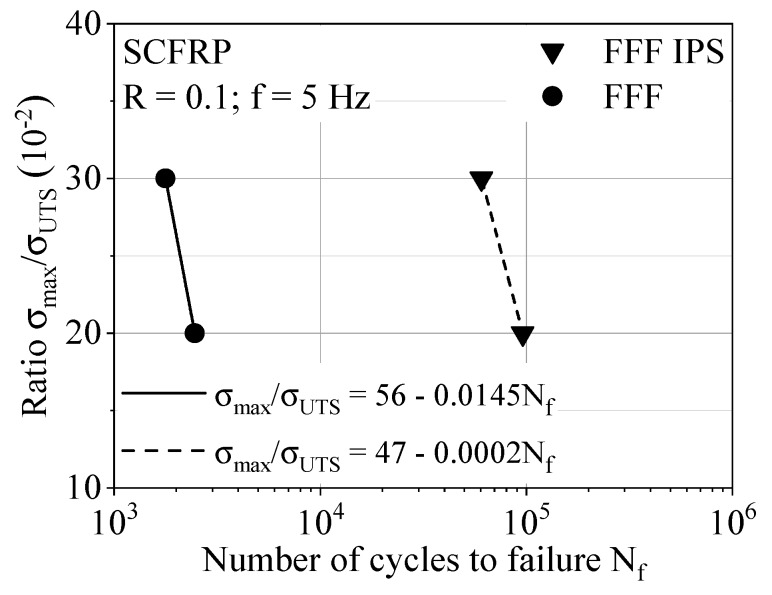
S/N curves for the tendency of lifetime estimation.

The basis for the cyclic tests was the quasi-static tests and the resulting ultimate tensile strength. The maximum stress level was selected depending on the ultimate tensile strength. The two stress levels at 20% and 30% of the ultimate tensile strength were chosen to qualitatively evaluate the influence of the process modification IPS on the lifetime. The aim of the experimental setup with cyclic loading was to identify a tendency of the lifetime with minimal experimental effort. The cyclic test data show no significant changes in temperature due to the cyclic load being at 5 Hz. The optical DIC device for monitoring the deformation behavior highlights the maximum strain perpendicular to the load introduction exactly between two layers. Figure 6 gives the results of the cyclic investigations; the abscissa shows the number of cycles to failure in logarithmic scaling, and the ordinate shows the maximum stress level in linear scaling. Despite the relative load as a function of the ultimate tensile strength, the use of IPS shows a significant effect on the material response under the cyclic tension–tension loading. The formulation of a linear equation describing the geometric relationship of two points in terms of the intercept of the axis and slope allows a first comparison for the lifetime performance. In particular, the difference in slope indicates a significant influence of the IPS on the material performance under cyclic loading and shows great potential for lifetime applications.

**Figure 7:**
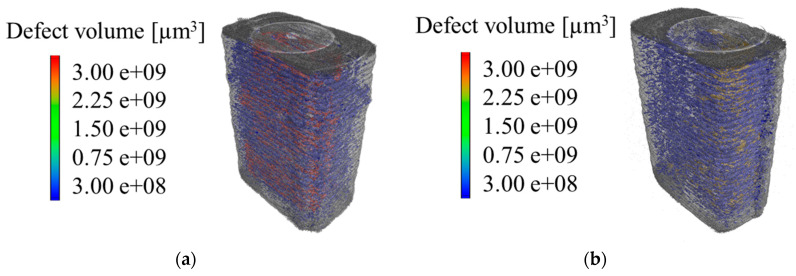
Representative micro-CT scans of the testing area: (**a**) FFF and (**b**) FFF IPS.

The micro-CT scans from the testing area of the respective specimens are shown in Figure 7. The standard defect analysis results in an absolute void volume content of 5.7% for the standard process FFF and 4.3% for the modified process FFF IPS. Qualitatively, the scans for the standard process FFF show a higher defect volume per void within the specimen. On the basis of this standard defect analysis and the measuring accuracy, the data do not allow any statement about significant differences in the defects and their distribution. Thus, the limitations of the standard defect analysis show a lack of informative value, which is to be extended by additional data processing.

**Figure 8:**
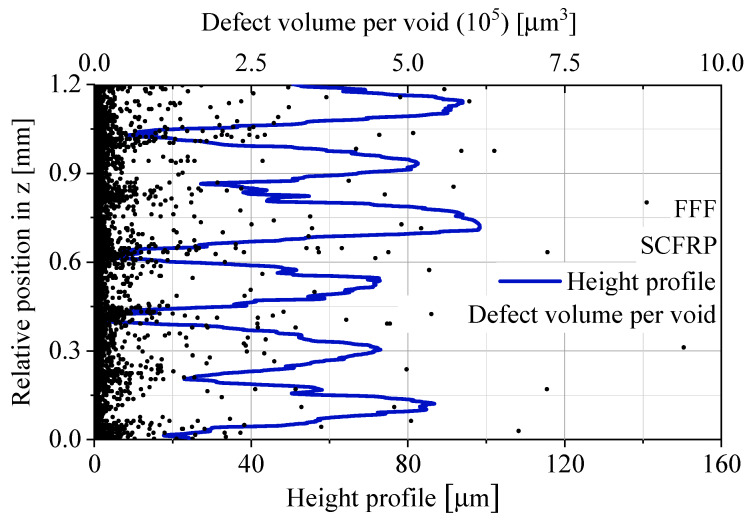
Void analysis of FFF based on micro-CT data, the void volume per void and the height profile vs. relative position in z.

**Figure 9:**
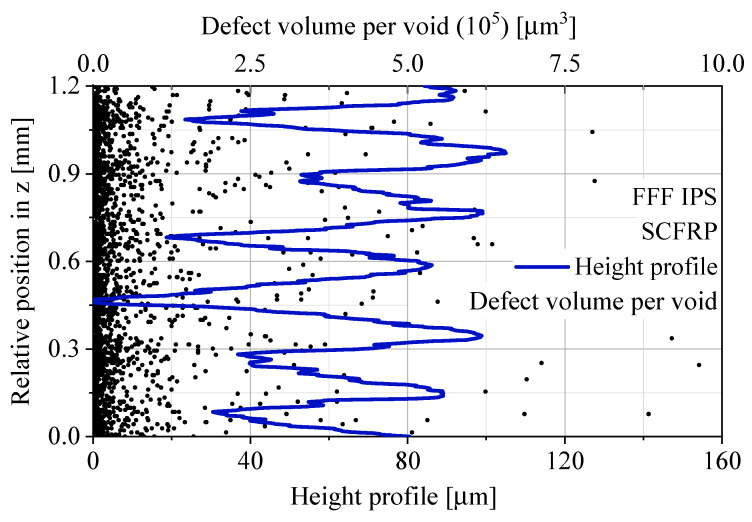
Void analysis of FFF IPS based on micro-CT data, the void volume per void and the height profile vs. relative position in z.

By processing the data of the defect analysis, the void distribution is observed more precisely. For this purpose, the three-dimensional micro-CT scans were sliced into two-dimensional layers with a height of 7 µm. The two-dimensional void areas in each layer are ideally calculated in a three-dimensional void volume per layer by multiplying it with the layer height of 7 µm. For each void in a layer, this results in an ideally void volume per layer for the defined layer height. Every individual void volume per layer was plotted as a function of the relative z position, which corresponds to the manufacturing direction z. In order to put the relative z position into a real context, the microscopically measured height profile of the specimen surface was superimposed on the void volume data. The height profile shows the typical macroscopic waviness of untreated additively manufactured FFF materials. The described data analysis is given in Figure 8 for the standard process FFF and in Figure 9 for the modified FFF IPS.

In the standard process FFF, voids accumulate at regular intervals of 200 µm, which corresponds to the manufacturing layer height in Table 2. The synchronization of the relative position in the z direction makes the position of the void accumulations visible. The increased number of voids appear for the standard process FFF in the interlayer contact zone. The modified process with the use of IPS does not show these accumulations. The distribution of void volumes per layer shows a quasi-homogeneous appearance without a pattern depending on the layer height of the manufacturing process.

The results of the experiments show the influence of the process modification IPS on the material performance and the void distribution. Bellehumeur et al. [22] highlighted that the formation of bonding in the FDM process is driven by the thermal energy of semi-molten material. The IPS provides an additional energy source that increases the thermal energy of the polymer surface. The higher temperature of the interlayer contact zone leads to an increased movement of polymer chains and thus to a higher interlayer bonding performance [23]. The quasi-static and cyclic experiments in this study show the same tendency. The non-destructive tests highlight a different void distribution within AM polymers due to IPS. The standard process FFF shows void accumulations between two layers resulting in reduced effective cross-sectional areas within the interlayer contact zone of FFF specimens. The modified process FFF IPS exhibits a quasi-homogeneous void distribution in the z direction. Therefore, the interlayer contact zone has an increased effective cross-sectional area compared to the standard process FFF. The different effective cross-sectional areas can lead to different stress concentrations, which have their origins at the different shapes of voids between two layers [24]. In addition, the results show various effects of the IPS on the material performance depending on the load type. The use of IPS improves the ultimate tensile strength by about 17%. Because of the high standard deviation, this improvement should be considered as a trend. In contrast, the results of cyclic investigations indicate a great improvement in the lifetime performance due to IPS. Compared to the quasi-static tensile tests, the tensile tests under cyclic loading are more structure-sensitive. The different bond formations, void characteristics and effective cross-sectional areas due to the IPS have a positive effect on the lifetime performance. In addition to the impact on the process development, the combination of testing technologies used for mechanical characterization can be summarized as a systematic approach. The methodology shows potential for detecting process-induced defects as well as structural differences of extrusion-based additively manufactured polymer.

## 5. Conclusions and Outlook

The obvious weakness of additively manufactured (AM) polymers due to material extrusion was deduced from the literature and selected preliminary tests in the manufacturing direction z. The standard process Fused Filament Fabrication (FFF) was adapted by an in situ infrared preheating system (IPS) to optimize the interlayer bonding performance of AM polymer. The evaluation of the process modification IPS requires a proper characterization of the interlayer bonding performance. Hence, a systematic approach to characterize the interlayer tensile strength is introduced. The destructive tests under quasi-static and cyclic loading were performed to estimate the material performance, and non-destructive testing methods like micro-computed tomography (CT) and 3D laser scanning confocal microscopy were executed for quality assessment.

A short carbon fiber-reinforced polymer (SCFRP) was used to investigate the process modification IPS for material performance and quality. The stress-based evaluation of the destructive tests is based on the initial cross-sectional area of the specimen. The innovative method for determining the initial cross-sectional area consists of the macroscopic measurement corrected by the surface roughness R_z_. The quasi-static tensile tests indicate a tendency for an improved interlayer bonding performance by using the IPS. The mechanical tests under cyclic loading highlight the opportunities to improve the lifetime performance of AM polymer with the IPS.

The absolute void volume content of the standard process FFF and the modified process FFF IPS is comparable. By processing the defect data, void accumulations of the standard process FFF are visible. The synchronization of the macroscopic height profile demonstrates the existence of void accumulations between the layers for the standard process FFF. The modified process FFF IPS shows a quasi-homogeneous void distribution for the manufacturing direction z. This results in a higher effective cross-sectional area in the interlayer contact zone.

The results of this study show the ability to characterize the interlayer bonding performance and optimize the overall material performance of AM polymer. Nevertheless, there is potential for improvement, both in the systematic approach and in the process modification. The current prototype of IPS consists of two long-wave ceramic radiators that are focused on the upper layer. The investigations, as well as the literature, show that the higher temperature level in the interlayer contact zone leads to better material performances. Nevertheless, the heat radiation has to be focused more precisely on the deposition spot, intensifying this effect of optimized material performances. The external heat source is also suitable for a defined cooling of the AM polymer. The crystallization of semi-crystalline thermoplastics can be influenced by defined cooling conditions. This is intended to further increase the material performance. This modification leads to a local material performance depending on process parameter gradients.

The systematic approach, which characterizes the interlayer bonding performance, is based on the interlayer tensile strength. The combination of destructive and non-destructive testing methods aims to separate process-induced defects for a proper characterization of the local material performance. In order to cover more application-oriented load types, the evaluation of the interlayer bonding performance will be extended with shear loadings. In comparison to different manufacturing directions, a more detailed consideration of the resulting surface roughness is taken. Due to different roughness parameters in the manufacturing directions, the use of an “as-built” surface does not enable a comparison. Thus, the effect of the surface roughness and the corresponding impact on the material performance has to be evaluated. Through quantification, a detailed comparative characterization of AM polymers is possible.

## Figures and Tables

**Table 1:** Selected material performances of CarbonX™ processed by FFF under tensile loading [3,21]. Layer height = 0.2 mm, Infill = 100%, Extrusion temperature = 260 °C, Manufacturing orientation according to ASTM F2921-11.

Material Characteristic	Unit	Variation	Values	Source
		XYZ	4540	[3]
		YXZ	2291	[3]
Young’s modulus	MPa	ZXY	1436	[3]
		Infill: ±45°	5075	[21]
		Infill: Concentric	3839	[21]
		XYZ	46.1	[3]
		YXZ	29.1	[3]
Tensile strength	MPa	ZXY	10.6	[3]
		Infill: ±45°	46.3	[21]
		Infill: Concentric	37.8	[21]

**Table 2:** Selected parameter gradients for the manufacturing processes FFF and FFF IPS. Manufacturing orientation according to ASTM F2921-11.

Parameter	Unit	CarbonX™
Nozzle diameter	mm	0.4
Extrusion bead	mm	0.5
Layer height	mm	0.2
Orientation	-	ZYX
Extrusion temperature	°C	260
Velocity	mm·s^−1^	10
